# Finding Your Identity and Partner in a Trade Mark? Consumption, Innovation and the Law

**DOI:** 10.1007/s40319-022-01229-z

**Published:** 2022-08-10

**Authors:** Jessica C. Lai, Janine L. Williams

**Affiliations:** 1 Associate Professor of Commercial Law, School of Accounting and Commercial Law, Wellington School of Business and Government, Victoria University of Wellington, Wellington, New Zealand; 2 Lecturer of Marketing, School of Marketing and International Business, Wellington School of Business and Government, Victoria University of Wellington, Wellington, New Zealand

**Keywords:** Trade marks, Brands, Identity, Personality, Partner, Innovation

## Abstract

Trade marks are not traditionally considered to be central to innovation because they do not need to be innovative to be protected. Instead, trade marks are used to indicate the source of products and services. Of course, if consumers could not determine the source, this would reduce the incentive for traders to compete through innovative products and services. Here, we argue that trade marks implicate innovation in yet another way. Namely, because consumption can be based on identity characteristics and personality traits of trade marks, which can result in consumer-brand relationships that are either or both identity-related and/or partner-like, companies have incentives to innovate in accordance with the identity characteristics and personality traits. Failure to do so can result in negative reactions from consumers and relationship break-ups, which impacts innovation selection and hence societal good. We explore the implications of this for trade mark law theory and practice.

## Introduction

Trade marks are not traditionally considered to affect innovation and be part of innovation policy. In contrast to patents and copyright, trade marks are not necessarily inventive or creative and we do not protect them to incentivise their creation. Of course, trade marks play a part in the innovation process as “badges of origin” to sell innovative products and services. Furthermore, trade marks can ensure repeat custom and income that can be re-invested in new and/or improved products and services. Within these framings, trade marks serve products and services.

Here, we argue that there is another means by which trade marks can implicate innovation. Namely, they can embody certain attributes and can thereby play a role in a consumer’s identity and expression of that identity. Furthermore, through being attributed anthropomorphic features, consumers can have partner-like relationships with brands. Within this dynamic, the trade mark (and brand) is often the thing of value and the innovation (product or service) serves the trade mark.^1^ That is, companies sometimes invest in new and/or improved products and services in order to maintain the value of the brand. The brand can be the thing that consumers want and this comes embodied within a product or service. This can shape what innovations are created and also whether they are commercialised.

In this article, trade marks are understood as an integral and central part of brands. While the brand is a broader concept,^2^ trade marks act as brand beacons (or identifiers) and it is unrealistic to draw stark delineations around trade marks vis-à-vis brands.^3^ Moreover, case law from the Court of Justice of the European Union and the United States has expanded trade mark protection to protect advertisement and investment around trade marks, thereby protecting the brand.^4^ The following part outlines existing ideas on the role that trade marks do or do not play in innovation. Part 3 analyses the consumer and marketing literature on trade marks and brands as identity builders and signals, as well as partner-like relationships that exist between consumers and brands. Part 4 discusses how the identity and partner-like relationships between consumers and brands implicate innovation with respect to trade marks. Part 5 examines the potential implications vis-à-vis trade mark law. The final part concludes and addresses the importance of re-framing the role of trade marks within innovation dynamics.

## Setting the Scene

**Fig. 1:**
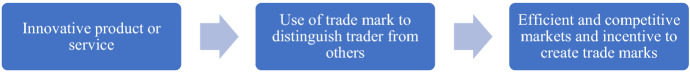
Trade marks merely serve innovation

As noted in the Introduction of this article, from an intellectual property law and policy perspective, trade marks are not conventionally considered to be a pivotal cog in innovation policy. Indeed, trade marks need not be innovative to be protected, which partly explains why one can potentially hold a trade mark in perpetuity – being unrelated to innovation, there is no need for a trade mark to fall into the public domain so that others can build upon it. Instead of being innovative, trade marks need only be capable of distinguishing one trader’s goods from another’s. It is even possible that a trade mark gains this capability through market use and marketing resource investment rather than through having inherent distinctiveness. Instead of being central to innovation, trade marks are theorised as having a core function as a “badge of origin” – signalling the source of a product or service.^5^ Law and economics theory states that the law protects this function (whether via registered trade mark systems or common law mechanisms, such as passing off or unfair competition) because it is in the consumer’s interest to quickly and correctly know the source of a product or service.^6^ This reduces consumer confusion and helps consumers distinguish between different products or services, which lowers transaction costs and allows for a more efficient market (see Fig. 1). In other words, trade marks are not so much considered to be about innovation per se, as about selling innovative products and services. Related to this, society wants companies to invest in trade marks so that consumers can distinguish products and services based on their source. This makes markets more competitive. Companies would not make this investment if others could imitate their marks.

Though trade marks themselves need not be creative (though they very well might be), they nevertheless play an intricate role in the innovation cycle. Companies have incentives to re-invest in new and/or improved products and services to remain competitive and continue to generate income. Yet, law and economics arguments for trade marks state that the incentive might be diminished if consumers could not distinguish between the products and services of different traders. Indeed, there might be little incentive to create high quality products if trade marks were not protected and another trader could confuse consumers about source.^7^

Furthermore, trade marks can facilitate re-investment in new and/or improved products and services. This can occur as trade marks can allow for consumer loyalty to build, because goodwill develops around them, which in turn ensures income.^8^ After all, goodwill is:

the benefit and advantage of a good name, reputation, and connection of business. It is the attractive force which brings in custom. It is the one thing which distinguishes an old established business from a new business at its first start. The goodwill of a business must emanate from a particular centre or source. However widely extended or diffused its influence may be, goodwill is worth nothing unless it has a power of attraction sufficient to bring customers home to the source from which it emanates.^9^

**Fig. 2:**
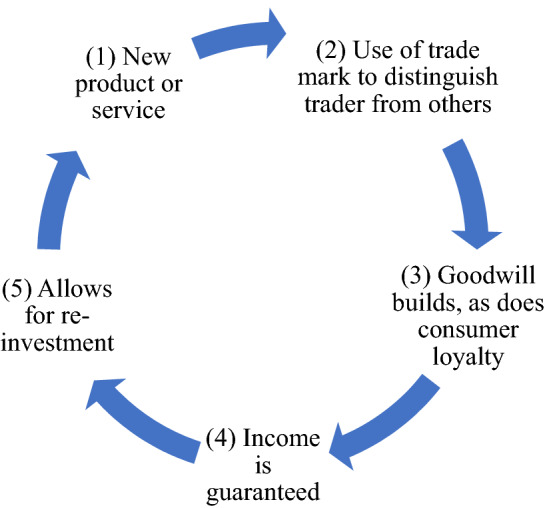
Trade marks facilitate re-investment in innovation

When this goodwill is associated with a brand it is termed brand equity – “a set of brand assets and liabilities linked to a brand, its name and symbol that add to or subtract from the value provided by a product or service to a firm and/or to that firm’s customers”.^10^ Goodwill and guaranteed custom allow a company to spread its fixed and research and development costs, which lowers the risk of investment in new and/or improved products and services.^11^ The knowledge that the company has consumer loyalty also reduces the risk of investing in new products and services, as the company’s reputation does not have to be newly established and there is a guaranteed consumer base (see Fig. 2).^12^ Additionally successful innovation can enhance perceptions of the trade mark and increase usage of the associated products and services.^13^ Thus, the cycle continues.

Another way to view this is that one can have a very inventive or creative product or service. However, without good branding and marketing communication, there will be little consumer demand and loyalty.^14^ The trade mark is an integral part of the brand. Marketing aims to increase awareness of the trade mark so that consumers can recognise and recall it (brand awareness), while simultaneously creating unique and positive associations necessary to generate demand and loyalty for the associated products and services (goodwill).^15^ Without consumer demand and loyalty, a company might not have the resources to invest in new and/or improved products and services. Indeed, it might not even recoup its initial costs. Thus, companies need “badges of origin” to distinguish and differentiate their goods and services, and to build up goodwill to ensure continued investment in their product.

The core function of trade marks as badges of origin is not dependent on inventiveness or creativity intrinsic to the mark. Instead, it relies upon individual and social meaning making, and strong, positive and unique associations between the mark, the product and the consumer, which build over time as value is co-created with and between consumers.^16^ This co-creation is important and we will touch upon it again throughout this article. Marketing communications play a role here as well. That is, trade marks develop meaning within a given social situation, become associated with this meaning, which over time forms a brand image. This is true even for marks that are inherently distinctive, as their meaning will still be socially contingent. In Steven Wilf’s words, with respect to trade marks, “the communicative sign is a placeholder for a robust but intangible cultural relationship between producer and consumer”.^17^ Beyond this social meaning making, the strong and unique associations contribute functional, experiential and symbolic benefits to the brand. Functional benefits solve a consumption problem – these are essentially utilitarian benefits. Experiential benefits meet consumer needs for pleasure, variety or cognitive stimulation, and symbolic benefits are associations with a particular self-image, role or membership in a group.^18^ To illustrate, a well-known sports brand may signal quality materials and workmanship, give the user pleasure through consumption, and symbolise that they are sporty. Such associations allow the generation and build-up of goodwill and facilitate consumer loyalty. Thus, social meaning making, along with strong positive and unique associations, allow a trade mark to gain the ability to act as a badge of origin.

At the same time, consumers get value from being able to quickly identify the source of products and services. They also gain from consuming the products and services. Yet, the value to a consumer does not just relate to the utilitarian use of a product or service and functional benefits per se. Consumers can perceive value in the way a product was made or service was provided (such as social or environmental standards) and the company behind it (such as whether it is innovative). However, these are credence qualities, which are characteristics of a product or service that a consumer cannot easily learn the truth about before or after purchase and consumption, regardless of how much experience that consumer has had buying that kind of product or service.^19^ Credence qualities may reflect corporate social responsibility, for example, whether an item of clothing is made by workers paid a living wage. It is not possible to know this by consuming the product. When it comes to credence qualities, there is an information asymmetry between manufacturers and consumers.^20^ Trade marks can signal information to bridge this information asymmetry.^21^ This is due to social groups associating certain aesthetic qualities with particular attributes, such as green as being environmentally friendly or pink as being feminine.^22^ It is also because a trade mark can come to embody certain qualities to a particular social group through social meaning making.^23^ For example, it is likely that consumers do not know exactly what the Fairtrade certification trade mark embodies (that is, the exact standards that the mark represents). Yet, they intuit what it expresses in a general sense.

So far, we have primarily discussed the communicative functions of trade marks at the point of purchase. However, because trade marks can embody certain characteristics, consumers can also gain value from the consumption of the trade mark and branding on the product or service. As alluded to above, there are emotional and self-expressive benefits of consuming trade marks, which are conferred by the fact that trade marks have market-constructed associations. As discussed in the following, social meaning making and the unique associations with trade marks contribute towards trade marks and brands being consumed to create, maintain and signal identity, and also to build consumer-brand relationships.

## Trade Marks as Identity Signals and Partners

As noted in the foregoing, value to a consumer vis-à-vis trade marks is not only determined by utilitarian means and based on the use of trade marks as badges of origin. Consumers also use trade marks to identify credence qualities about the products and services. Furthermore, consumers can get experiential and symbolic value from consuming the trade marks and brands themselves. As a result, some brands have goodwill that is disproportionately larger than the intrinsic value of the products or services to which they relate.^24^ For example, while Apple is backed by high quality products, its brand has value beyond this. This is partly related to what it signifies, for example, being exciting.^25^ At the same time, there are brands that come with a premium, but that are not necessarily backed by proportionately high-quality products. In such cases, the consumer is often paying for the brand and what it signals or communicates, not the quality of the product.^26^ “Trade mark merchandising”, for example, of a sports team logo, exemplifies this, as consumers are more interested in the mark and the product is ancillary.^27^ In addition, with these marks it is often unlikely that a consumer would believe that the mark indicates source. To illustrate, when one buys a Manchester United (an English Premier League football team) T-shirt, it is very unlikely that this is in the belief that the product comes from the team. This, amongst other examples of trade mark protection, led Mark McKenna to conclude that, “[s]uccinctly stated, modern trade mark law is industrial policy intended to protect brand value”.^28^

The consumption of trade marks and brands is not limited to merchandising. To illustrate, a consumer might be embarrassed by “budget” packaging, perhaps because symbolically it makes him/her look “cheap” or poor, so buys a more expensive product that is not proportionately higher in quality to avoid this embarrassment. As a further illustration, the signalling or communicative effect of brands explains in part why some people buy counterfeit products even though they know they are fake – the quality is not central, the brand and what it symbolises are. Finally, that the brand has value beyond the product or service to which it is attached is exemplified by the fact that consumers will continue to pay more for brand-name/originator medicines after cheaper generics enter the market.^29^ As generic medicines must be bioequivalent to originator medicines to obtain regulatory market approval, there is no rational or utilitarian reason to continue to pay a premium for the brand-name/originator medicine. Yet, certain consumers will stick with the brand-name/originator, perhaps out of loyalty or habit, or because they associate it with the pioneering innovation and believe the products are more effective and safer.^30^

Thus, Barton Beebe opined that the physical thing upon which a trade mark is attached has less and less of a role in sustaining cultural meaning.^31^ Beebe explained that it is well understood that “consumers communicate with each other by the objects they consume. Of late, however, commodity culture has begun to unburden itself of the object language of material commodities. The trademark system has developed as an alternative language of consumption, and its development has been rapid indeed”.^32^

The result of trade marks being used as devices that communicate via consumption is that “[t]he culture industries – and what industries aren’t? – have long sold trademarks as commodities in their own right”.^33^ Beebe continued that much trade mark doctrine, thus, constitutes “systems of rules designed to facilitate the commodification – indeed, the ‘industrial production’ of social distinction”.^34^ In other words, the language of consumption has developed and is used as means of social differentiation, because society demands means to socially differentiate, and create social structures and hierarchies.^35^

That consumers can gain from consuming trade marks themselves partly results from the fact that they signal more than source.^36^ As well as indications of quality and credence attributes, trade marks can embody and reflect human characteristics. Because of this, consumers can use trade marks and brands to build, maintain and signal attributes that reflect their actual and idealised image of self, role identities and group memberships.^37^ That is, trade marks and brands can be employed to create and preserve an identity, and “can serve as identity-expression vehicles”.^38^ This applies to services, as well as products.^39^ Notably, the flipside of the coin is that absence of consumption can also reflect one’s identity.^40^ Consumer behaviour, from where to shop to what to buy (and where not to shop and what not to buy), is thus partly driven by the expression of self,^41^ including drivers that pertain to before and after the point of purchase. To illustrate how human characteristics affect purchasing behaviour, impulse buying occurs at a greater rate for identity-related products than non-identity-related products.^42^ As a further illustration, consumers who identify as global citizens tend to use global brands to signal this.^43^ In contrast, country of origin branding can be used to reinforce national identity.^44^

A variety of kinds of identity-related attributes can be signalled. This includes lifestyle choices, such as being athletic, outdoorsy or indoorsy, or socially or environmentally conscious. Trade marks can also signal relational characteristics, for example, being individualistic or familial, as well as group affiliation, such as political, vocational, national or sports team affiliations. Finally, as discussed further below, trade marks can signal personality traits, such as being extroverted or introverted, “proper”, free-spirited or rebellious, tough or soft, sexy or cute. The brand identity attributes can be closely related to means of creation and maintenance of self and collective identity including social structures and hierarchies.

Of course, what is signalled depends on the audience. As discussed above, meaning is not merely individualistic, but socially constructed within specific social locations.^45^ Thus, while a brand might signal one thing in a certain situation, it might signal something different in a different context. Indeed, some marks might have strong associations in one context, but have no meaning in another. To illustrate, while O2 (a wireless telecommunications company in the United Kingdom) might engender certain sentiments in the United Kingdom (possibly different in various parts of the country), the trade mark has essentially no meaning in New Zealand, where O2 does not trade.

In addition, meaning is non-static but is continuously re-made by the trader, the consumer and others, creating a consumer culture around brands.^46^ That is, meaning making is not unidirectional. It is dependent on the trader’s market behaviour, which includes the trade mark, the product or service (which can also carry social meaning, for example, through shape and colour), pricing, distribution strategy and associated statements and marketing communication from the trader. Meaning is also dependent on consumers, namely, how they interpret and associate the trade mark and brand,^47^ and whether they decide to invest in the brand with their attention, time and money.^48^ This, in turn, is dependent on society, as meaning making is socially contingent.^49^ Note that the consumer and the public are contributing to, and investing in, both the meaning of trade marks, as well as the goodwill attached to them.^50^ Such an understanding of meaning runs contrary to the static, unidirectional and almost passive view that the law and economics approach takes to trade mark law, which presumes that trade marks only convey information about source and quality, and once that information has been obtained by a passive recipient,^51^ it does not adjust.^52^ Furthermore, the law and economics approach ignores the contribution and investment that consumers and the public make to the meaning of marks and their goodwill.

Consuming things that align with one’s self-image affects one’s well-being, as well as self-verification and self-enhancement.^53^ Exactly what one consumes is made complicated by the fact that different aspects of one’s identity might conflict, and which attribute dominates is situational. One’s identity is dynamic and in any given moment its expression depends on situational affordances and constraints.^54^ In other words, which aspect of one’s identity is expressed depends on the exact context and whether that aspect is cued or restricted. To illustrate, a consumer might identify as being very environmentally conscious, but also as someone who makes prudent financial choices. Perhaps correct product placement and an “on sale” sign might cue the consumer to pay more for a green product. On the other hand, perhaps shopping with a parent who raised the consumer to “count pennies” might restrict the consumer from buying a more expensive green option.

Recent studies have focused on ethical and moral considerations and how branding can affect consumption in this regard. For example, branding indicating that the product or service is environmentally friendly or made within socially acceptable standards, such as with respect to fair pay, safe working conditions, or equality regardless of gender or sexuality. Social and environmental concerns can form part of one’s identity and can affect one’s consumer behaviour, for example, perceived value in a product or service can increase, as can willingness to switch products/services and intention to purchase.^55^

Trade marks and brands not only signal certain social characteristics, they can also be prescribed anthropomorphic features, giving them personality. For example, a brand might be described as competent or warm, sincere, trustworthy, moral, deceitful, exciting, sophisticated or rugged.^56^ Consumers often refer to brands in a way that ascribes them agency.^57^ As a result of this anthropomorphism, consumers can form emotional attachments to trade marks,^58^ with consumer-brand sentiments varying in intensity from brand passion/love through indifference to brand hate.^59^ At the more extreme positive end, consumers can relate to brands in a partner-like fashion.^60^ The bond that is created goes beyond simple brand loyalty with some degree of interdependence, as brands are perceived to have human qualities.^61^ It is also possible that consumers use brands as relationship substitutes.^62^ However, where the brand and its characteristics are perceived with indifference, a relationship is likely avoided altogether.^63^

Like human-human relationships, consumer-brand relationships can be complex and changing, and involve relational concepts like falling in love, romance, loyalty, relationship management and maintenance, and betrayal.^64^ In addition, consumers’ individual attachment styles can play out in these relationships, affecting which brand personalities a consumer attaches to, as well as the level or kind of attachment.^65^ Furthermore, power dynamics can develop, with consumers forming master-slave relationships with brands, as either the former or the latter.^66^ Consumers have been found to participate in these relationships with differing levels of commitment (of time, sacrifice, money and energy) to maintain the relationship. Such commitment may be influenced by brand trust, or the extent to which one can rely on the brand’s functional utility, as well as the perceived quality of a brand as a relationship partner.^67^ Thus, it is not surprising that brand personality dimensions of sincerity and competence are the strongest predictors of brand commitment, while excitement and ruggedness are the weakest.^68^

When things are going well, these relationships contribute positively to the goodwill associated with the trade mark and brand through increased satisfaction and loyalty, and consumers are more likely to engage with them, spread positive word-of-mouth about them, and to forgive their mistakes.^69^ These positive outcomes have been found to be most likely when the brand personality is sincere rather than exciting.^70^ In contrast, as discussed below, negative consumer experiences can result in avoidance and revenge-seeking behaviour against the trade mark.

Aware that brands can have personalities, companies use brand personalities strategically to differentiate their products and services and to appeal to their desired target segment.^71^ In light of the connection between brand personalities and the brand-consumer relationships, companies may also purposefully modify the brand personality should they wish to change relationship partners.^72^ More often than not, however, once a successful position is secured companies will act to strengthen the brand personality they have created due to the strategic value of maintaining their relationship with their target consumers and the associated goodwill they have generated.

## What Does This Have to Do with Innovation?

Because trade marks and brands develop images and personalities, and because consumers employ them to build, maintain and signal identity, the products and services on which trade marks are attached can be viewed as servicing the brand-identity-consumer dynamic. Analogously, when consumers relate to brands in a manner similar to human-human relationships, products and services upon which trade marks are placed are integral to the image and relationship retention and maintenance.

Companies that have such trade marks that are related to identity and/or part of a consumer-brand partner-like relationship have incentives to innovate with their products and services to maintain the identity signals and personality attributes.^73^ The fact that consumers associate with brands that are congruent with their identity, and brands have personality traits that allow for partner-like relationships between brands and consumers, means that an innovation that creates sudden incongruity or change in personality can have negative, even violent, outcomes. Like a romantic relationship, love can turn to hate. When consumers hate brands, they may avoid and/or speak badly about them, even going so far as to become anti-brand activists and encouraging others to boycott them.^74^ While consumers are more forgiving of the brands with which they have strong and positive relationships, changes can occur that result in dissolution of the relationship. The trade mark may be associated with a transgression or the consumer may simply grow apart from the brand, perhaps due to changing values, leading to “brand divorce”.^75^ There is potential for innovation that is incongruent with the brand to be viewed as either a transgression or a value-based change, and consumers may feel betrayed. This may result in consumers avoiding the brand, ending the relationship, and possibly even seeking revenge. The stronger the relationship, the faster it falls apart and the longer the consumer’s negative reaction and revenge.^76^ Furthermore, the more self-relevant the relationship (i.e. related to the consumer’s actual or idealised identity), the more likely the consumer will take retaliatory action, which may be anything from giving poor reviews to illegal behaviour such as theft, threats and even vandalism.^77^ At the same time, the stronger the relationship, the more likely that the consumer will be receptive to an attempt by the company to recover the relationship.^78^ Forgiveness will depend on the nature of the transgression.^79^

**Fig. 3:**
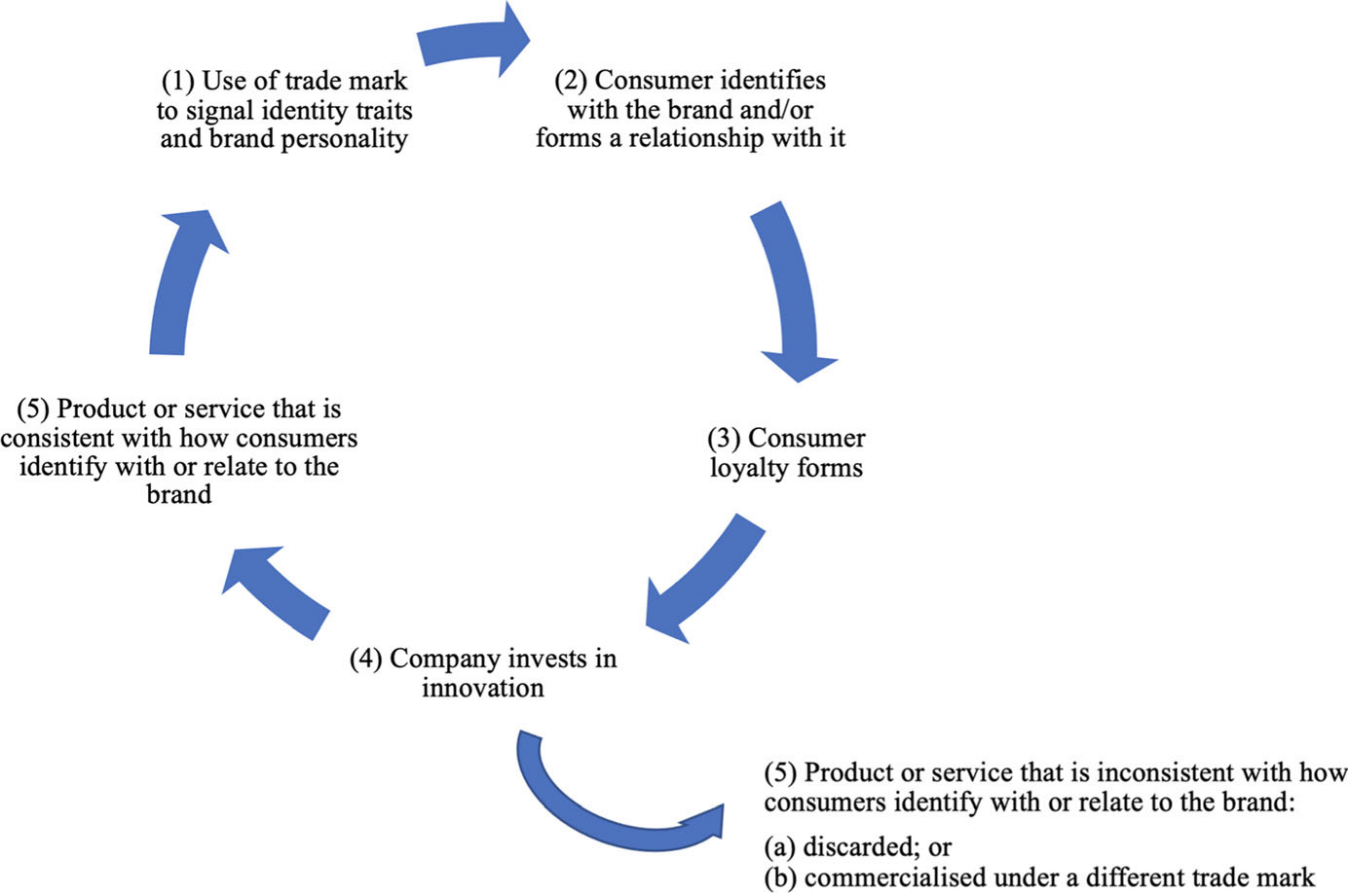
Innovation typically facilitates the maintenance and retention of identity and personality features of trade marks

Therefore, to avoid the breakdown of the brand-consumer relationship and consumer retaliation, companies have an incentive to invest in new and/or improved products/services in a way that retains identity congruity and brand personality (see Fig. 3). The extent to which a company feels this incentive would depend on the degree to which the trade mark has identity-related signals and/or anthropomorphic personality, the nature of this personality and associated consumer-brand attachment. Strategically, the innovation should reinforce the brand or broaden its meaning in a positive way amongst the target audience. Otherwise, it may change brand image and hence jeopardise the nature of the relationship. The stronger the brand personality and brand attachments, the greater the incentive to do so. In this way, the trade mark imposes limitations on which innovations are pursued.^80^

To illustrate, trade marks relating to budget bread are likely to signal price-based value. A budget bread company will invest in branding that creates this image or personality. As budget bread is priced as cheaply as possible to attract a certain market – one that is likely more concerned about cost than branding, one would expect any innovation to be directed at reducing costs and hence maintaining or lowering the price rather than prioritising innovation that may increase costs. In contrast, one can imagine that a brand like BMW is quite the opposite. BMW can be used to build, maintain and signal identity typically related to status – the exact identity depends on the model and features.^81^ BMW has the personality traits of being precise, successful and powerful and is largely masculine.

Innovations or changes that are incongruent with the brand might have to be marketed under a different trade mark, where possible. For example, in 1994, BMW acquired Mini, which had historic associations of being small and economical – quite incongruous with the classic associations of BMW. Hence, rather than subsuming the Mini into the BMW brand, BMW maintained the separate brand (adopting a “House-of-Brands” strategy). It leveraged Mini’s historic associations and positioned Mini as fun, sporty, fashionable and stylish.^82^ Such consistency is the underlying premise of integrated marketing communication, which facilitates the formation of consumer-brand relationships and enhanced brand equity. As discussed further below, not all companies have the resources to develop and maintain a House of Brands.

Limitations imposed by the existing brand personality and customer relationships extend beyond the type of innovation to the degree of innovation pursued. Companies make decisions regarding innovation efforts in relation to what industry they are in, their brand and market position (e.g. whether one is the market leader, follower, or niche player), and their orientation to the market (whether they are driving the market or market-driven). Typically, market leaders have been found to pursue game-changing radical innovation, whereas market followers tend to innovate only incrementally.^83^ However, the degree of innovation is tempered by the brand personality. When a company is deciding whether and how to innovate, it must consider the effects of leveraging its existing brand equity versus potential negative effects for current offerings and consumers. Put another way, brands face a tension between enhancing their relevance through innovation, whilst, at the same time, maintaining consistency with their brand identity. For example, a mature and trusted brand such as BMW, with a sincere and competent brand personality, may be less able to pursue market-driving radical innovation in comparison to a more exciting brand such as Tesla. The company behind BMW has incentives to invest in innovation in their cars to maintain and retain the different identity signals and personality traits. Failure to do so, for example, releasing a car that is technically faulty, not aesthetically pleasing in the right way, or incompatible with the consumer’s values or lifestyle, might mean that a consumer no longer identifies with BMW or wishes to end the relationship with the brand. Inconsistency that confuses or alienates consumers could even result in brand hate and the associated behaviours could be destructive for the goodwill associated with the trade mark. Thus, brand personality can restrict the scope of innovation adopted. That said, if the brand personality is exciting, consumers will expect the company to be more innovative. As noted, one likely expects Tesla to offer market-driving radical innovation. To further illustrate, in 2010, Apple’s customers criticised it for only innovating incrementally, making minor adjustments to their products, which was not seen as congruent with their brand personality.^84^

The relationship between a trade mark and an innovation can be synergistic and positive in many respects. As the trade mark represents the image in the consumer’s mind of the firm’s value offering, changes to this offering, such as through innovation, can contribute positively to these associations with the brand. A brand that innovates may be seen as more entrepreneurial and competent than one that does not. Innovation also increases the visibility of the brand, attracting attention and generating excitement. At the same time, when consumers have a strong self-brand connection, they are more likely to counter-argue with, or discount negatives associated with the brand, and adopt, or at least accept, innovation.^85^ Consumers will also be less resistant to risks associated with radical innovation when the brand personality is high in openness to change, as compared to if the brand is conservative. By way of example, in a study on consumer acceptance of artificial intelligence in self-driving cars, consumers indicated higher intention to adopt autonomous cars under Tesla (open) than Ford (conservative).^86^

The importance of fit between a brand and an innovation poses some issues for trade mark practice and theory. In a situation where there is a low fit between the innovation and the trade mark, consumers may not gain access to a promising new offering. This is because the launch of an innovation may be delayed or never happen at all to avoid incongruence between the trade mark and product. This could be negative if the innovation is something socially, economically or environmentally valuable, for example. To illustrate, a trade mark and brand may have the traits of being risky and individualistic. An innovation that would make the product safer or perhaps more environmentally friendly might not be brought to market, because it would be incongruent with the trade mark, which could be negative for the relationship between the trade mark, product and consumer. Thus, the trade mark’s characteristics, and how consumers relate to the trade mark, can shape innovation both in directing creation and also commercialisation.

As alluded to above, larger entities can deal with brand-innovation incongruity by creating a new trade mark and brand, and maintaining a House of Brands. We noted above that BMW exemplifies this – it has sufficient resources to create and maintain such a House of Brands. However, small- and medium-sized entities may not be in a position to develop and maintain a new brand and associated identity. This means that trade mark and innovation incongruence resulting in innovations being held back may be particularly salient for small- and medium-sized entities, which may steer away from launching certain innovative products and services due to potential negative outcomes if their existing trade marks are used.

For larger organisations with very strong brands the disincentive may relate simply to an association risk. While the innovation may cannibalise existing offerings, it could also create a backlash against the brand’s existing products.^87^ For example, Apple may have the capability to innovate lower-cost alternatives. However, it may not do so partly because its consumers would switch between its more expensive to its more affordable products, reducing Apple’s profit line. Apple might also not do so due to the strength of its current brand and the potential backlash for the parent trade mark/brand if it were to introduce lower-cost alternatives, even if Apple were to create and use a sub-brand.

## Should This Implicate Trade Mark Law?

Acknowledging the practical role that trade marks play in directing innovation and commercialisation should be significant for trade mark theory, as it affects our reasoning of why entities have trade marks and the ways in which consumers gain from them. It also impacts our understanding of the drivers of innovation and commercialisation. There are many possible implications of this for trade mark law. To illustrate, if we know that the identity and anthropomorphic features of trade marks can affect innovation and what is commercialised, we could strategically define what one can get a trade mark over in order to shape innovation and what is brought to market. Alternatively, it could affect the application of legislative provisions relating to immoral or offensive marks. This might, for example, be to channel innovation away from what is anti-social or detrimental for the environment. Or, in the opposite direction, it might be to encourage certain features in trade marks to promote certain innovations or certain consumer behaviour. For example, it might affect the trade marks that we allow for pharmaceutical companies, which need to be trustworthy but also capable of introducing a radical innovation. The world has observed the importance of this with Covid-19 vaccines.

We note that this could prove challenging due to the social and non-static nature of meaning making.^88^ The same thing can have different meanings to different social groups, or to the same social group across time. In this vein, without further regulation, entities could use things such as marketing communications to shape meaning making in their favour, thereby circumventing any legislative or policy intent. It is also arguable that trade mark law could be a relatively blunt tool for directing innovation – potentially giving broad-stroked nudges towards or away from certain innovations, but without any specificity. Put another way, changes in trade mark law alone would potentially be insufficient to shape innovation, particularly in the long term, as marketers and marketing communications would adapt. A suite of measures would be required, including, for example, within general consumer law or specifically regarding the kind of marketing communications that can be made for certain products or services (such as nicotine, or therapeutic products).

Any operationalisation of the relationship between trade marks, identity, brand personality and consumers would have to factor-in potential effects on competition. As discussed above, law and economics theory holds that trade marks are pro-competitive, as they force traders to compete through their products and services rather than imitating one another. However, as we have shown, in reality, it is also possible that traders use brand personalities to differentiate their products and services, thereby making themselves more unique and consequently reducing their need to compete through their products and services.^89^ Indeed, it is common to compete on brand alone in crowded markets. In addition, that brand personalities can restrict what is innovated and/or whether an innovation is brought to market could further reduce competition. This is particularly true for companies with brand personalities that are more sincere and conservative, which are less likely to compete through market-driving radical innovations. This tugs at a deeper issue, namely, that it is unrealistic to view trade marks as mere badges of origin that are purely pro-competition. Like patents and copyright, trade marks can be, and sometimes are, anti-competitive because of what they can symbolise and their associated brand personalities, which affect consumer loyalty, innovation and commercialisation.

A question that arises is whether the kind of innovation that goes into trade mark creation, and brand development and maintenance, benefits society in a way, or to a degree, that justifies this anti-competitiveness. It is theorised that the anti-competitive potential of patents and copyright is justified by the incentive that the protection generates to create inventions and works. Law and economic theory states that copyright and patents address a market failure, as – in the absence of intellectual property protection – the ability to copy one’s ideas and creations would result in people inventing and creating less, and less invention and creation would be negative for society. It is not clear whether this is similarly arguable for trade mark protection. Put another way, patents and copyright are presumed to be anti-competitive, but this is considered acceptable because they incentivise invention and creation. In contrast, trade marks are presumed to be pro-competitive. However, we have shown here that this is not necessarily the case. Can this be justified?

When we acknowledge that trade marks can and do affect competition and innovation, sometimes negatively, this brings into question whether trade marks should be capable of being held in perpetuity, restricting all use of the mark, as a trade mark, in the course of trade. If we conceive of trade marks narrowly as mere badges of origin, reflecting goodwill, then it makes sense that the owner can continue to renew the registration for as long as he/she pleases, and has the exclusive right to use the mark in the course of trade. However, if trade marks are more than this trader-goodwill-consumer link, also being anti-competitive and affecting innovation, then perhaps we need to re-think the owner-user/public interface. Put another way, this should implicate the degree to which we allow third parties to use registered trade marks, and also whether and how trade mark registration can be lost.

A discussion on third-party use of trade marks necessarily requires some explanation on the scope of owners’ rights. Traditionally and theoretically, trade marks protect against consumer confusion by ensuring trade marks can perform their core function as badges of origin. However, in practice, trade marks signal more than origin, including a myriad of messages, characteristics, attributes and personalities, and often these signals have significant value, as discussed throughout this article. Thus, it is perhaps unsurprising that trade mark protection has expanded beyond protecting against consumer confusion. As indicated in the introduction, what constitutes infringement has expanded to protect well-known marks against third parties using the mark in a way that causes “dilution”, or takes “unfair advantage”, of the distinctive character or the repute of the mark. There need not be any consumer confusion. Dilution includes “blurring” by impairing the distinctiveness of a mark, and “tarnishing” by harming the reputation of the mark. Martin Senftleben has noted that protection from “dilution” constitutes protection of the investment made in creating a brand image, which is quite different from the traditional rationales of trade mark protection of preventing consumer confusion, and ensuring market transparency and fair competition.^90^ Similarly, being able to prevent third parties taking “unfair advantage” can protect investment and advertisement, which is arguably protection of the communicative aspects of trade marks.

The protection of the communicative aspects of trade marks, other than of origin, is highly questionable. This is in part because of the fact that the meaning of trade marks is co-created by consumers and the public,^91^ as highlighted throughout this article. Jessica Litman, Steven Wilf, Rochelle Cooper Dreyfuss and Deborah Gerhardt have separately argued that the co-creation of the communicative aspects of trade marks means that third-party use of those aspects should be allowed. Litman stated that we need to keep in mind that brands are not just created by their producers, but also by the public, as “[t]he argument that trade symbols acquire intrinsic value – apart from their usefulness in designating the source – derives from consumers investing those symbols with value for which they are willing to pay real money”.^92^

The law protects the trade mark owners’ investment and advertising in the brand (image and meaning), but ignores the consumers’ and public’s investment. Yet, Wilf has argued that the co-creation means that consumers and the public also *author* trade marks in a “joint interpretive enterprise”.^93^ He further stated: “By associating a symbol with an object, the public contributes to the authorship of trademarks”.^94^ After all, an inventor invents an “invention”, an author authors a “work”, but it is use of a mark in trade that allows for the generation of goodwill. In his words, “Good will is an identification created by the public”.^95^ “The producer affixing a symbol might be called primary meaning while secondary meaning embodies the idea of public association. This association takes place in the midst of a market where linguistic exchange parallels the transfer of goods. Both the producer and the consuming public are joint authors”.^96^ That the law recognises that marks can gain a secondary meaning (allowing descriptive terms to be registered), or that marks can be struck from the register through “genericide”, confirms that the public contributes to meaning making.^97^

Wilf argued that, as the public is a co-author of a trade mark, the public should also have rights to the trade mark. However, trade marks do not naturally fall into the public domain after a set period of time, unlike patents or copyright. Thus, Wilf contended that trade marks should be limited in other ways from the outset, with an internally constructed public domain resulting from the co-authorship.^98^ He stated:

Association means authorship and context points to meaning. Within trademark doctrine lies the material to construct a public domain. During the moment of associational creation, the public permits certain kinds of trademark privileges. The public never alienates symbols or words from the Lockean commons because to do so would be to lose touch with a collective self.^99^

That is, as a co-author of a trade mark, the public gives trade mark owners certain rights, but does not wholly give up its own rights to those marks and their signals.

Furthermore, protecting investment and advertising, as well as use that does not cause consumer confusion, seems unfair when one considers that consumers and the public also invest in brands, and that their investment allows for continued marketing campaigns.^100^ Litman noted that trade mark owners “built up all the mystique with their customers’ money and active collaboration”.^101^ She argued that the co-creation of a brand is a “collaborative undertaking; the investment of both dollars and imagination flows both ways”, and there is no justification for only one party (the trade mark owner) to reap all the benefits of that collaboration.^102^ Instead, she argued that “the icons that embody the persuasive force of those brands … should properly be viewed as collectively owned”.^103^

Finally, Dev Gangjee has opined that the protection of the signal function of trade marks beyond origin presumes that reputation, image and identity are “bounded and static enough to be appropriated”.^104^ Yet, as discussed throughout this article, meaning is co- and re-created, situational and changing. In light of this, Gangjee stated that “we may be reaching for the wrong metaphors when characterising brands in legal discourse. It is instead possible to reconceive of brands as a commons or elements of the public domain”.^105^

There is, of course, a deeper question regarding the presumption that there is ownership at all in signalling beyond ensuring consumers are not confused. Litman argued that there is no justification for protecting against use that does not cause consumer confusion. She stated that we have to place competition at the heart of decisions around what trade mark law protects, seeing as trade mark protection is premised upon being pro-competitive.^106^ In her words:

in devising the rules of trade symbol law, we need to keep our eyes, first and foremost, on competition. The enforcement of trade symbol rights is not costless. As the realm of protection expands, it necessarily does so at the expense of competition. Competition, though, is the basis for the rationale underlying any protection of trade symbols. If we do not want to encourage producers of different products to compete with one another for consumers’ dollars, then we do not really need to protect trade symbols at all.^107^

In line with Litman and Wilf, Gerhardt has argued that the consumer co-investment and co-creation of meaning and value should affect the law, in particular, infringement disputes and the public’s right to use marks.^108^ Trade marks are typically considered to be less anti-competitive than patents or copyright, as others can simply use different marks to sell their products or service. However, this ignores the communicative and identity functions of trade marks to consumers and the public – they need the marks to communicate. And should they not be able to use the marks to communicate when they are co-constructors of their meanings? In this vein, Gerhardt argued that more salience should be given to consumer interests vis-à-vis how they use marks, to reflect a public interest in using marks in a communicative sense (as tools of information). Gerhardt argued that consumers should get a return on their investment by being allowed to use marks to express information. This would be, for example, for non-deceptive use of marks in keyword advertising, through a safe harbour for reference tools (such as dictionaries and encyclopaedias, and in a defence of “cultural dilution” (applicable if the general public has invested a mark with a new cultural significance).^109^

Gerhardt’s suggestion of a defence of “cultural dilution” is consistent with Cooper Dreyfuss’ call for a doctrine of “expressive genericity”. Cooper Dreyfuss critiqued trade mark law vis-à-vis third parties using trade marks to communicate something beyond the identity intended by the trade mark owner.^110^ More specifically, she argued that there should be a doctrine of “expressive genericity”, where owners of marks should not be able to enforce their trade marks when they are used by third parties to express meaning beyond either origin or that invoked by the owner, if the trade mark is rhetorically central to the usage. She stated that it should not be trade mark owners that garner the value of this “surplus” expressive genericity that is created by consumers and the public.^111^

Cooper Dreyfuss noted that allowing the public to communicate with trade marks with the meanings that they created would not affect companies’ incentive to “create” trade marks, as they have economic incentives to develop “nice symbols” to sell their products and services,^112^ and to “develop a vocabulary with which to conduct commerce”.^113^ The same argument could be made to allow the public to use trade marks to communicate meaning that it co-created with the trade mark owner. Senftleben has taken this a step further. Like Cooper Dreyfuss, Senftleben argued that the expansion of trade mark protection into the communicative functions of trade marks should be met with defences similar to those found in copyright law that protect interests in freedom of expression.^114^ In addition, Senftleben has noted that, rather than being concerned about whether there is sufficient incentive for traders to create brand images, we should be concerned “that seductive lifestyle messages conveyed by a trademark distract from a product’s genuine qualities, thereby rendering consumers’ buying decisions less objective and depriving the trader with the objectively best offer of corresponding market success”.^115^ That is, we should be concerned about the incentives traders have to create marks and marketing communications that convey meaning unrelated to the quality of products or services.

Bringing this together, we should be concerned with the seduction of marketing communications that do not relate to information asymmetry, the protection of this communication to the detriment of consumers who have co-created meaning and value of marks, and the negative impact that this can have by stifling innovation contrary to that meaning. If we believe that consumers should consume based on information about the origin and quality of a product or service, that the public has a right to meaning and value that it co-created, particularly in light of freedom of expression, and that innovative developments should be based on something other than brand identity, then trade mark law should only protect against use of trade marks in the course of trade that affect signals relating to origin and quality.

Even more provocative, perhaps we should re-think grounds for losing one’s registration. For example, perhaps trade mark owners should lose the registration if the mark loses the ability to signal origin and quality, and instead only signals other meaning. Typically, it is possible that one loses a registration due to a mark becoming generic. That is, that the mark becomes descriptive of a general class of products or services, thus losing its ability to signal origin and quality. The standard for “genericide” is high and very few marks are de-registered on this ground. What we are proposing here is different in that it would not be about the mark becoming descriptive of a general class of products or services, but that it communicates only something other than origin and quality. We imagine that this would be rare.^116^ However, it would serve as a further reminder that the primary purpose of trade marks is to serve as badges of origin, allowing customers to associate a good/service and its quality with a trader. In this vein, David Vaver has argued that, if consumers are not using trade marks to choose rationally between different products and services, the protection of those marks becomes questionable vis-à-vis our underlying justification for trade mark protection.^117^

## Conclusion

The protection of trade marks is traditionally understood as necessary to ensure that consumers can distinguish between goods and services from different traders, allowing for an efficient and competitive market (see Fig. 1). Trade marks have not customarily been considered to be central to innovation because trade marks do not need to be innovative per se – they need only be capable of distinguishing one trader’s good from another’s, potentially through market use rather than inherent distinctiveness. Thus, trade marks have conventionally been viewed as playing a service role to innovative products and services. That is, innovation occurs and this is followed by the use of a trade mark to ensure consumers can identify the source of the innovation.

More recent law and economics literature recognises that trade marks do indeed play a role in innovation. This is in part because, when traders are distinct to consumers, traders must compete through their products and services. Put another way, failure to protect trade marks would result in consumers not being able to distinguish between the products and services of different traders, which would reduce the incentive to invest in creating innovative and high-quality products and services. Furthermore, it has been recognised that trade marks play a part in innovation because they allow for goodwill to build and consumer loyalty to form, which guarantees income that can be used to invest in new and/or improved products and services (see Fig. 2). In any case, per either the traditional understanding or more recent law and economics approach, the trade mark serves the product or service.

In this article, we have argued that trade marks implicate innovation beyond merely forcing companies to compete through their products or services, or allowing for re-investment in new and/or improved products and services. Trade marks also play a more active role in directing the development of new products and services. They do so through their brand identity signals and anthropomorphic personalities, which afford trade marks the potential to have a salient role in consumer identity and consumer-brand relationships. For some trade marks and brands, where the identity effect is high and/or the consumer-brand relationship is strong, the trade mark and brand become the thing of value and the identity signals and personality traits are what have to be maintained and retained. In such cases, innovation in new products and services serves this maintenance and retention, and ultimately the value of the trade mark and brand (see Fig. 3).

This re-framing is by no means absolute and clear cut. Depending on the kind of product or service, not all trade marks and brands have strong identity signals or personalities, and it might not be an aim of a trader to have these. There is also an obvious chicken-and-the-egg problem, as usually trade marks are preceded by a product or service. Furthermore, the initial product or service itself might be part of the creation of the identity features and personality traits. Perhaps these issues can be overcome by recognising that the starting point is not salient. What is important is that there are companies that have (or wish to have) trade marks that consumers use to build, maintain and signal identity and/or develop consumer-brand partner-like relationships, and this affects innovation dynamics for these companies.

Moreover, the re-framing is important as it allows us to recognise value in trade marks and brands per se, as well as their integral part in not only allowing for further innovation, but also impacting and directing the innovation. This is relevant for the innovation of products and services to gain, maintain and retain customers. It is also pertinent if a company wishes to purposefully change the demographic of its average consumer, as they should be aware of the potential fall-out of their products or services altering identity characteristics and personality traits related to the trade mark.

Trade mark law and policy should not only protect traders but also consumers, and they are theorised as doing this by signalling authenticity via linking innovations to traders, and ensuring that competitors compete through their products and services. The reframed perspective drawn in this article highlights how the consumer may be impacted by the relationship between the trade mark and innovation beyond this link. The trade mark may decrease consumer resistance to radical innovations when it is consistent with the innovation. However, it may restrict access to promising innovations that are inconsistent. This could have anti-competitive outcomes. Thus, while existing theories hold that trade marks are pro-competitive through the products and services upon which they are placed, we have shown here that this is not necessarily the case. Overall, this reframed perspective contributes to how we theorise trade marks and their effect on innovation and competition, which should impact the scope of the protection of trade marks. On the basis of consumers’ co-creative role in brand meaning associated with trade marks and the cultural significance that can be acquired, it is important that trade mark protection is limited to origin and quality. It should not restrict the use of symbolic aspects of brands that are the result of co-creative engagement. If the law aims to enhance healthy competition and acknowledge consumer agency, then it must adhere to this purpose and we should narrow the scope of protection.

## Footnotes

1 Lury (2009), p. 201.

2 *See e.g*. Schroeder (2009), p. 161; and Lury (2009), p. 223.

3 Senftleben (2009), p. 48, who states “trademarks can be qualified as the focal point of communication with consumers”.

4 Case C-487/07 *L’Oréal SA v Bellure NV* [2009] ETMR 55. For analysis, *see* Gangjee (2013), p. 29. For an analysis of United States’ case law on the expansion of trade mark protection to protect marketing and “persuasive elements”, *see* Litman (1999), pp. 1725–1728; Cooper Dreyfuss (1990), p. 397.

5 E.g. *Intercity Group (NZ) Ltd v Nakedbus NZ Ltd* [2014] 3 NZLR 177 [98], stating “in New Zealand the essential function of a trade mark is as a badge of origin”; *E & J Gallo Winery v Lion Nathan Australia Pty Limited* [2010] HCA 15 [43].

6 Landes and Posner (1987). For critique of the search-cost theory of trade marks, *see* McKenna (2012); Dogan (2018); Dinwoodie and Janis (2007). Note that McKenna’s historical analysis of trade mark law shows that trade mark law was not originally about consumer protection, but to protect producers from illegitimate diversion of sales – protecting against consumer deception was ancillary to this; McKenna (2007).

7 Chon (2017), p. 303; Rogers (1949), p. 176. Stronger trade mark protection, beyond just protecting against source confusion, can result in products being protected, which can make trade mark law more like copyright law or patent law; Beebe (2004), p. 661; Chung (2018).

8 Gangjee (2021), p. 192. Gangjee critically analyses attempts to empirically link trade mark registration numbers and “innovation”, partly by critiquing causality, highlighting the various reasons for trade mark registrations that have little to do with innovation, as well as criticising the nebulous meaning of “innovation” as trade marks might be closely connected with some kinds of innovation, but possiby not innovation in the patent law sense.

9 *CIR v Muller & Co’s Margarine Ltd* [1901] AC 217 (UK House of Lords), pp. 223–24.

10 Aaker (1991), p. 15.

11 Gangjee (2021).

12 Note that these law and economics arguments for trade mark protection based on a consumer’s ability to distinguish source do not explain the move towards protecting trade marks as property, particularly through dilution laws; *e.g*. Bently (2008), p. 3; Beebe (2004), p. 621.

13 Brexendorf et al. (2015).

14 Dogan (2018), p. 269. *See e.g*. Smith and Hansen (2002), p. 371: “the value of IP does not stand in isolation, but depends on how well the organization can convert it into value for the customer. No matter how brilliant the thinking and insight of a patented concept or technology, if it cannot be converted into something that delivers a tangible benefit to someone, it has no value”. Sponsorship and licensing can also affect the value of a mark; Ng (2009), p. 236.

15 *See e.g*. Senftleben (2009), who refers to this as “sign programming”.

16 Arnould and Thompson (2005); Wilf (1999), p. 8 (who states that, after a producer associates a sign with an object, “that association is recognized and invested with meaning by the public as an interpretive community”); Gangjee (2013); Peukert (2019), pp. 1179–1182; Merz et al. (2009); Ramaswamy and Ozcan (2016).

17 Wilf (1999), p. 2.

18 Park et al. (1986).

19 Darby and Karni (1973). One could also refer to such qualities as “Potemkin qualities”, which are attributes that cannot be verified through testing. Credence qualities can be contrasted with search or experienced qualities, such as the price of a good or the taste of it, respectively; Nelson (1970). *See also* Griffiths (2013).

20 Akerlof (1970).

21 Katz (2010), pp. 1563–65; Bently (2008), pp. 6–16. *Cf*. Desai (2014), who argues that this ignores the behavioural economics that consumers are not rational, but make purchases based on emotional and psychological grounds, such as following the herd. More so than normal trade marks, certification trade marks are designed to overcome information asymmetries; Lai (2021).

22 *E.g*. Beebe (2004), p. 624, who describes trade mark doctrine as the “language of commodities”.

23 Schroeder (2009).

24 Litman (1999), p. 1728; Senftleben (2009), pp. 68–70.

25 Aaker (1997); Maehle et al. (2011), p. 295.

26 Peukert (2019).

27 McKenna (2007), p. 1911.

28 *Ibid*., p. 1916.

29 Wan (2018); Gangjee (2021), pp. 202–204; Bronnenberg et al. (2015); Dutfield (2021).

30 Possibly there is some status quo bias; Thaler and Sunstein (2009), pp. 37–39; and Shrank et al. (2009).

31 Beebe (2004), p. 657.

32 *Ibid*., p. 703.

33 *Ibid*., p. 624.

34 *Ibid*., p. 624.

35 *Ibid*., p. 703.

36 On the hedonistic value of trade mark consumption, see Dogan (2018), pp. 278–80.

37 Berger and Heath (2007); Kleine et al. (1993); Belk (1988); Mittal (2006).

38 Wheeler and Bechler (2021), p. 6. *See also* Solomon (1983); Elliot and Wattanasuwan (1998); Munteanu and Pagalea (2014).

39 *See e.g*. Underwood et al. (2001).

40 Gerhardt (2010), p. 465.

41 Leigh and Gabel (1992); Levy (1959); Woodruffe-Burton and Wakenshaw (2011); Hogg and Michell (1996).

42 Dittmar and Bond (2010).

43 Strizhakova et al. (2011).

44 Swaminathan et al. (2007).

45 Elliot and Wattanasuwan (1998).

46 Schembri et al. (2010); Arnould and Thompson (2005).

47 Wilf (1999).

48 Gerhardt (2010), pp. 449–467.

49 Fournier and Alvarez (2019).

50 Wilf (1999), p. 17.

51 Gerhardt (2010); Gangjee (2013), p. 49.

52 *Cf*. Desai (2014).

53 *E.g*. Swann and Read (1981); Aguirre-Rodriguez et al. (2012).

54 Oyserman (2009a); Oyserman (2009b); Kirmani (2009), p. 273.

55 Shaw et al. (2000); Confente et al. (2020); Singh (2016). Note that “virtue signalling” can also affect consumer behaviour, where individuals might feel that they have to purchase a certain way (*e.g*. more environmentally friendly) to look good to their peers. On virtue signalling, *see* Stollznow (2020).

56 Aaker (1997); Aaker et al. (2010).

57 Kervyn et al. (2012).

58 Malär et al. (2011).

59 Park et al. (2013).

60 Fournier, Breazeale and Avery (2015); Fournier (1998); Alvarez and Fournier (2016); Fetscherin et al. (2019).

61 Fournier (1998).

62 Halloran (2014).

63 Park et al. (2013).

64 Fournier (1998).

65 Swaminathan et al. (2009).

66 Alvarez and Fournier (2016), p. 132; Swaminathan et al. (2009).

67 Jahn et al. (2012), pp. 451, 452 and 453; Albert et al. (2013).

68 Eisend and Stokburger-Sauer (2013), pp. 205, 211 and 214.

69 Park et al. (2010); Batra et al. (2012), p. 12; Hegner et al. (2017).

70 Aaker et al. (2004).

71 Plummer (1985); Elliot and Wattanasuwan (1998).

72 Fournier (1998).

73 Lury (2009), pp. 209–210, stated that “productive innovation does not derive or emerge from innovation in the production process. Instead it is produced in practices of simulation or behaviour modelling – that is, through qualification trials in which products (often prototypes) are experimentally tested in relation to the goal or aim of reaching a target consumer market. In short, innovation does not originate in the production process, but rather emerges in relation to meeting the ‘needs’ of the market, where those ‘needs’ are interpreted in terms derived from marketing information about the consumer”.

74 Fetscherin (2019).

75 Sussan et al. (2012).

76 Grégoire et al. (2008); Fetscherin (2019).

77 Johnson et al. (2011).

78 Grégoire et al. (2008).

79 Joireman et al. (2016).

80 Beverland et al. (2010).

81 Johansson-Stenman and Martinsson (2006).

82 Eisend and Stokburger-Sauer (2013), p. 215; Simms and Trott (2016).

83 Beverland et al. (2010).

84 *Ibid*.

85 Swaminathan et al. (2007).

86 Casidy et al. (2021).

87 Joshi, Reibstein and Zhang (2009), pp. 936–37.

88 In the US, there might be free speech implications. *See e.g*. *Matal v Tam* (2017) 137 S Ct 1744.

89 Note that trade mark provisions and case law that protect against comparative advertising are also anti-competitive. Similarly, certain shape, colour, smell, taste and sound trade marks might also be anti-competitive.

90 Senftleben (2009), pp. 50 and 57. *See also* Ng (2009), pp. 235–237.

91 Cova and Dalli (2009); Gangjee (2013).

92 Litman (1999), p. 1730.

93 Wilf (1999), p. 45.

94 *Ibid*., p. 1.

95 *Ibid*., p. 12.

96 *Ibid*., p. 33.

97 *Ibid*., p. 32.

98 *Ibid*., p. 7.

99 *Ibid*., p. 45.

100 *Ibid*., p. 17.

101 Litman (1999), p. 1730.

102 *Ibid*., p. 1733.

103 *Ibid*., p. 1733.

104 Gangjee (2013), p. 46.

105 *Ibid*., p. 57.

106 Litman (1999), pp. 1731–1735. *Cf*. Schechter (1927).

107 Litman (1999), p. 1735.

108 Gerhardt (2010), pp. 464–467.

109 *Ibid*., pp. 469–499.

110 Cooper Dreyfuss (1990). *See also* Schroeder (2009) on the way brands affect culture with meaning.

111 Cooper Dreyfuss (1990), pp. 402–403, 407.

112 *Ibid*., p. 409.

113 *Ibid*., p. 399.

114 Senftleben (2009), pp. 60, 62–63.

115 *Ibid*., p. 59. *See also* Vaver (2009), pp. 171, 197.

116 On the fact that marks are often not indicators of source, *see* Ng (2009), p. 235.

117 Vaver (2009), p. 198.
